# Consensus-based technical recommendations for clinical translation of renal diffusion-weighted MRI

**DOI:** 10.1007/s10334-019-00790-y

**Published:** 2019-11-01

**Authors:** Alexandra Ljimani, Anna Caroli, Christoffer Laustsen, Susan Francis, Iosif Alexandru Mendichovszky, Octavia Bane, Fabio Nery, Kanishka Sharma, Andreas Pohlmann, Ilona A. Dekkers, Jean-Paul Vallee, Katja Derlin, Mike Notohamiprodjo, Ruth P. Lim, Stefano Palmucci, Suraj D. Serai, Joao Periquito, Zhen Jane Wang, Martijn Froeling, Harriet C. Thoeny, Pottumarthi Prasad, Moritz Schneider, Thoralf Niendorf, Pim Pullens, Steven Sourbron, Eric E. Sigmund

**Affiliations:** 1 Department of Diagnostic and Interventional Radiology, Medical Faculty, University Dusseldorf, Moorenstr. 5, 40225 Düsseldorf, Germany; 2 Department of Biomedical Engineering, Istituto di Ricerche Farmacologiche Mario Negri IRCCS, Bergamo, Italy; 3 MR Research Centre, Department of Clinical Medicine, Aarhus University, Aarhus, Denmark; 4 Sir Peter Mansfield Imaging Centre, University Park, University of Nottingham, Nottingham, NG7 2RD UK; 5 Department of Radiology, Cambridge University Hospitals NHS Foundation Trust, Addenbrooke’s Hospital, Cambridge, UK; 6 Translational and Molecular Imaging Institute and Department of Radiology, Icahn School of Medicine at Mount Sinai, New York, NY USA; 7 Developmental Imaging and Biophysics Section, UCL Great Ormond Street Institute of Child Health, London, UK; 8 Imaging Biomarkers Group, Department of Biomedical Imaging Sciences, University of Leeds, Leeds, UK; 9 Berlin Ultrahigh Field Facility (B.U.F.F.), Max Delbrueck Center for Molecular Medicine in the Helmholtz Association, 13125 Berlin, Germany; 10 Department of Radiology, Leiden University Medical Center, Leiden, The Netherlands; 11 Department of Diagnostic, Geneva University Hospital and University of Geneva, 1211 Geneva-14, Switzerland; 12 Department of Radiology, Hannover Medical School, Hannover, Germany; 13 Die Radiologie, Munich, Germany; 14 Department of Radiology, Austin Health, The University of Melbourne, Melbourne, Australia; 15 Department of Medical Surgical Sciences and Advanced Technologies, Radiology I Unit, University Hospital “Policlinico-Vittorio Emanuele”, University of Catania, Catania, Italy; 16 Department of Radiology, Children’s Hospital of Philadelphia, Philadelphia, PA USA; 17 Department of Radiology and Biomedical Imaging, University of California San Francisco, San Francisco, CA USA; 18 Department of Radiology, University Medical Center Utrecht, Utrecht, The Netherlands; 19 Department of Radiology, Hôpital Cantonal Fribourgois (HFR), University of Fribourg, 1708 Fribourg, Switzerland; 20 Department of Radiology, Center for Advanced Imaging, NorthShore University Health System, Evanston, IL USA; 21 Department of Radiology, University Hospital, LMU Munich, Munich, Germany; 22 Comprehensive Pneumology Center, German Center for Lung Research, Munich, Germany; 23 Ghent Institute for Functional and Metabolic Imaging, Ghent University, Ghent, Belgium; 24 Department of Radiology, University Hospital Ghent, Ghent, Belgium; 25 Department of Radiology, Center for Biomedical Imaging (CBI), Center for Advanced Imaging Innovation and Research (CAI2R), NYU Langone Health, New York, NY USA; 26 Department of Radiology, University Hospital Tuebingen, Tübingen, Germany

**Keywords:** Biomarker, DWI, ADC, IVIM, DTI

## Abstract

**Objectives:**

Standardization is an important milestone in the validation of DWI-based parameters as imaging biomarkers for renal disease. Here, we propose technical recommendations on three variants of renal DWI, monoexponential DWI, IVIM and DTI, as well as associated MRI biomarkers (ADC, *D*, D*, *f*, FA and MD) to aid ongoing international efforts on methodological harmonization.

**Materials and methods:**

Reported DWI biomarkers from 194 prior renal DWI studies were extracted and Pearson correlations between diffusion biomarkers and protocol parameters were computed. Based on the literature review, surveys were designed for the consensus building. Survey data were collected via Delphi consensus process on renal DWI preparation, acquisition, analysis, and reporting. Consensus was defined as ≥ 75% agreement.

**Results:**

Correlations were observed between reported diffusion biomarkers and protocol parameters. Out of 87 survey questions, 57 achieved consensus resolution, while many of the remaining questions were resolved by preference (65–74% agreement). Summary of the literature and survey data as well as recommendations for the preparation, acquisition, processing and reporting of renal DWI were provided.

**Discussion:**

The consensus-based technical recommendations for renal DWI aim to facilitate inter-site harmonization and increase clinical impact of the technique on a larger scale by setting a framework for acquisition protocols for future renal DWI studies. We anticipate an iterative process with continuous updating of the recommendations according to progress in the field.

**Electronic supplementary material:**

The online version of this article (10.1007/s10334-019-00790-y) contains supplementary material, which is available to authorized users.

## Introduction

Diffusion-weighted (DWI) magnetic resonance imaging (MRI) has been shown to provide differentiated information on the microstructure of kidney tissue. Furthermore, significant efforts have been made to adopt DWI-based parameters as an MR biomarker for functional renal imaging [1–6]. However, to successfully translate the research results of renal DWI to clinical practice, there are still some challenges to overcome. Firstly, acquisition protocols vary between research groups and reflect local practice and expertise. Secondly, patient preparation, data post-processing and image analysis are not standardized, with several approaches being used by different research groups. As has been recognized by other consortium efforts [7–9], our motivation behind prioritizing standardization of these processes is the generation of reliable MRI biomarkers that are ready to be broadly utilized in multi-site studies. When achieved, the data generated from standardized study protocols will sufficiently increase the evidence base to determine threshold values for DWI-based parameters, to differentiate between renal pathologies. Histopathological correlation should also continue to be performed to ensure diagnostic validation of the MRI biomarkers. With the aim to move toward a standardization and to facilitate the validation of DWI-based parameters as a renal MRI biomarker, an international, multidisciplinary group of renal imaging researchers with experience and/or ongoing work in renal DWI was recently formed as part of the ‘PARENCHIMA’ (the European Cooperation in Science and Technology) COST action (www.renalmri.org).

As a first step in this endeavour, Caroli et al. [10] published a review and statement paper reflecting the current state of research to assess diffuse renal pathology by renal DWI. The work summarizes the acquisition protocols used in human renal DWI studies up to August 2017 (172 studies) involving both healthy subjects and patients with renal disease. It highlights the large diversity in acquisition protocols, patient preparation and image post-processing techniques, as well as the lack of “gold standard” for the measurement of in vivo renal DWI. This diversity of acquisition protocols across studies has led to a variability of acquired quantitative renal diffusion parameters, which is summarized in the detailed supplement material of the review [10]. Therefore, a further mission of the PARENCHIMA initiative is building consensus on renal DWI acquisition protocol, patient preparation and post-processing techniques.

In this work, a consensus on recommended acquisition protocol for renal DWI was formed consistent with the consensus-building goals of the Delphi process [11–13]. The design of the surveys for the consensus building was informed by a literature review (extending the prior review until November 2018) that aimed to identify which acquisition parameters had the most impact on DWI measurements. For the development of the recommendations, the three most common variants of renal DWI techniques used in the literature were considered: (1) monoexponential model with parameter apparent diffusion coefficient (ADC); (2) biexponential model or IVIM (intravoxel incoherent motion) model with the parameters water diffusion in the tissue (*D*), flowing fraction (*f*) and pseudodiffusion (D*); and (3) diffusion tensor imaging (DTI) with mean diffusivity (MD) and fractional anisotropy (FA). All three variants of renal DWI techniques aim to estimate a diffusion constant of water in tissue. However, in all models this diffusion constant is named differently (ADC, *D*, and MD). ADC quantification methods considering a non-Gaussian DWI signal behavior are not covered in these recommendations given their more preliminary stage of investigation and are not deemed as ripe for standardization as the other methods described above. We summarize the three common renal DWI approaches and associated quantification methods below.

### Monoexponential ADC

This quantification model for diffusion-weighted MRI is the most popular due to its simplicity and modest acquisition requirements. The monoexponential ADC model assumes a uniform Gaussian displacement distribution of the water molecules corresponding to a monoexponential diffusion-weighted signal decay of the MR signal. The computation of the monoexponential ADC is based on the Stejskal–Tanner equation [14]:

1 $$\frac{{S_{\text{b}} }}{{S_{0} }}\; = \;e^{{ - b{\text{ADC}}_{\text{mono}} }}$$

where *S*_b_ is the diffusion-weighted signal intensity, *S*_0_ is the signal intensity without a diffusion weighting (*b* = 0 s/mm^2^)*, b* is the diffusion weighting strength (in s/mm^2^), and ADC_mono_ is the apparent diffusion coefficient of water within the observed image voxel.

For renal tissue, the monoexponential model is known to be insufficient to describe the diffusion-weighted signal decay, with IVIM effects occurring at low *b* values (< 200 s/mm^2^) [15] and non-Gaussian effects possibly occurring at high *b* values (> 800 s/mm^2^). However, as a single parameter estimation, the monoexponential model provides relatively robust ADC and requires only moderate signal-to-noise ratio on DWI.

Given the contrast effects mentioned above, the estimated ADC is strongly dependent on the choice of selected b-values [15, 16] and no consensus exists with regard to the choice and number of *b* values in a renal DWI acquisition protocol. Taking into account Eq. (1), a set of minimum two *b* values is enough to reach a stable diffusion signal [16, 17] for the quantification of ADC. However, most authors prefer to describe the diffusion signal decay more precisely by including more *b* values in the acquisition protocol. Considering possible anisotropic diffusion, it is common practice to measure the *b* values in several orthogonal directions during the ADC acquisition [15, 16].

### Intravoxel incoherent motion (IVIM)

First described by Le Bihan et al. [18] in 1986 the IVIM model is another option to interpret the physiological underpinning of the diffusion signal. Since the initial studies in human subjects by Muller et al. in 1998 [19] and later by Thoeny et al. in 2006 [20] showing the potential of the IVIM model to interpret diffusion signal in the kidney, this quantification has been demonstrated to improve the representation of the diffusion-weighted signal in renal tissue compared to the ADC [21–23].

IVIM considers the diffusion signal originating from two different compartments. One compartment reflects the slow thermal diffusion in the tissue (*D*), hindered or restricted by local microstructure. The second compartment considers the fast molecule movement associated with incoherent flow in the microvasculature or renal tubules that mimic random water motion assuming that many vessel and tubules orientations are present within the voxel (quantified by the pseudodiffusion, D* and the flowing fraction, *f*).

This method of quantification utilizes a biexponential decay, describing the overall diffusion-weighted signal as the sum of the diffusion and flowing components:

2 $$\frac{{S_{\text{b}} }}{{S_{0} }}\; = \;\left( {1\; - \;f} \right) e^{ - bD} \; + \;fe^{{ - bD^{*} }}$$

where *S*_b_ is the diffusion-weighted signal intensity*, S*_0_ is the signal intensity without a diffusion weighting (*b* = 0 s/mm^2^)*, b* is the diffusion weighting strength (in s/mm^2^), *D* is the water diffusion in the tissue (slow component), D* is the pseudodiffusion (fast component), and *f* is the flowing fraction.

To quantify IVIM parameters, a minimum of four *b* values are needed to determine all unknown parameters in Eq. (2), which typically extends the acquisition time in comparison to the monoexponential ADC. Furthermore, there is no universally accepted algorithm yet to calculate IVIM quantitative parameters. In many studies, a so-called “segmented fitting” or “2-step” approach is used to calculate the IVIM parameters (2), due to its extended stability and faster fitting [24–27]. In the “segmented fitting”, a threshold *b* value is defined to separate flowing from diffusion effects (microcirculation-induced decay assumed negligible above this threshold). However, although *D* is more stable in the “segmented fitting”, than in others, the estimates of *f* and D* can be biased depending on threshold choice. More recently, Bayesian probability-based fitting methods have been explored, with or without fixing of the pseudodiffusion coefficient (this has shown higher precision/accuracy, and low inter-subject variability [28]).

Other, more complex, extended IVIM models can be found in the literature that aim to incorporate more characteristics of functioning renal tissue into the signal description. Three compartment models include an additional component taking into account multiple sources of intravoxel incoherent motion, e.g., due to the glomerular flow [29, 30], vascular vs. tubular flow, or residual fat signal [31]. Other extended models combine IVIM with diffusion anisotropy for a more comprehensive description of both structural and microcirculation features [32, 33]. These models are mentioned here solely to indicate current research frontiers as they require further investigation before they can be pursued in the context of consensus standardization.

### Diffusion tensor imaging (DTI)

Measurement of the directional dependence (anisotropy) of apparent diffusion in tissue microstructure provides a marker of that tissue’s integrity and thereby its clinical function. Diffusion tensor imaging (DTI) quantitatively measures and maps the anisotropy imposed on water diffusion by a tissue’s microstructure.

For DTI analysis, diffusion-weighted signals along several diffusion directions are acquired and fit to a 3 × 3 symmetric tensor model [34, 35].

3 $$\bar{D}\; = \;\left( {\begin{array}{*{20}c} {D_{xx} } & {D_{xy} } & {D_{xz} } \\ {D_{yx} } & {D_{yy} } & {D_{yz} } \\ {D_{zx} } & {D_{zy} } & {D_{zz} } \\ \end{array} } \right)$$

where $\bar{D}$ is the symmetric diffusion tensor with elements *D*_ij_ determined by the linear set of equations generated by the set of ADC measurements along each diffusion gradient direction [36] $\hat{g}$:

4 $${\text{ADC}}_{n} = \widehat{{g_{n} }}^{\dag } \cdot \bar{D} \cdot \widehat{{g_{n} }}\; = \; \mathop \sum \limits_{ij} g_{i,n} g_{j,n} D_{ij} .$$

More generally, all gradients (imaging and diffusion weighting) can be taken into account by computing the full b-matrix of their diffusion weighting:

5 $$- \ln \left( {\frac{S}{{S_{0} }}} \right)\; = \mathop \sum \limits_{ij} b_{ij} D_{ij} ,$$

$$b_{ij} = \mathop \int \limits_{0}^{\text{TE}} k_{i} \left( t \right)k_{j} \left( t \right)dt,$$

$$k_{i} \left( t \right)\; = \;\mathop \int \limits_{0}^{t} \gamma G_{i} \left( {t^{{\prime }} } \right)dt^{{\prime }} .$$

The eigenvalues of this tensor describe the maximal, intermediate, and minimal diffusion values, with eigenvectors reflecting their corresponding orientation. The primary eigenvector, associated with the largest eigenvalue, indicates the orientation of maximal diffusion.

6 $${\text{MD}}\; = \;\frac{1}{3}\left( {\lambda_{1} + \lambda_{2} + \lambda_{3} } \right),$$

where MD or mean diffusivity is the average of the diffusion coefficients and $\lambda_{1} ,\;\lambda_{2} ,\;\lambda_{3}$ are the eigenvalues.

Another parameter, called fractional anisotropy (FA), reflects the amount of diffusion directivity in DTI studies (0 = complete isotropy, 1 = complete anisotropy) and is calculated by

7 $${\text{FA}}\; = \;\sqrt {\frac{3}{2}} \; \times \;\frac{{\sqrt {\left( {\lambda_{1} - \overline{MD} } \right)^{2} \; + \;\left( {\lambda_{2} - \overline{MD} } \right)^{2} + \left( {\lambda_{3} - \overline{MD} } \right)^{2} } }}{{\sqrt {\lambda_{1}^{2} + \lambda_{2}^{2} + \lambda_{3}^{2} } }},$$

where again $\overline{\text{MD}}$ is the average of the diffusion coefficients, a DTI-specific ADC equivalent and $\lambda_{1} ,\;\lambda_{2} ,\;\lambda_{3}$ are the eigenvalues.

Several studies have demonstrated that DTI provides powerful biomarkers of diffusion isotropy in the cortex and anisotropy in the renal medulla [15, 16, 22, 37–43]. This behavior is consistent with the known structural organization of medullary constituents such as the tubular loops of Henle, collecting ducts, and vascular vasa recta, which have an inward radial pattern toward the renal pelvis.

As with many diffusion biomarkers, FA and MD depend on the number and magnitude of the applied *b* values [38, 42, 44]. As diffusion anisotropy is a key target of DTI, acquisition of multiple diffusion directions (minimally 6) is required for tensor computation. However, while some studies of diffusion direction choice in renal DTI have been performed supporting at least 12 directions [45], determination of an optimal number or choice of *b* values and directions for renal DTI, analogous to comprehensive efforts in the brain [28] or muscle [46], has not yet been performed.

## Materials and methods

### Literature review and data extraction

To justify the motivation for the standardization process, assess the state of the renal DWI literature, and provide input to subsequent recommendations, we summarize reported DWI biomarkers from a wide range of prior renal DWI studies assessing diffuse renal diseases. These efforts build upon reviews and meta-analyses that have aimed to understand the variability of reported renal diffusion biomarkers in the literature [4, 15, 23].

A systematic review and analysis of the literature (using the same search criteria in PubMed as previously used by Caroli et al. [10], but extending those until November 2018) was carried out.

Specifically, papers were categorized according to their protocol and quantification scheme as either monoexponential DWI (113 studies), DTI (40 studies), or IVIM (41 studies). From each paper, we extracted protocol parameters including full *b* value ranges, repetition times (TR), echo times (TE), number of gradient directions and field strength. The distribution of *b* value ranges was extracted for each DWI model for visualization. Additionally, DWI biomarkers were also extracted for cortex, medulla, and whole kidney (as available in each study), reporting values in healthy adult controls. For each *b* value range, the maximum and average *b* values were also computed. Monoexponential DWI studies provided ADC values [20, 21, 43, 47–61], DTI studies provided MD and FA values [29, 32, 33, 38, 42, 45, 62–71] and IVIM studies provided *D*, *f* and D* values [20, 21, 28, 29, 32, 42, 43, 54, 55, 68, 71–78].

Following data extraction, correlations were computed in healthy volunteers only via Pearson correlation coefficients with the following protocol parameters: (1) TR; (2) TE; (3) average *b* value; (4) maximum *b* value; (5) transverse relaxation factor T2 = exp (− TE/T2*t*); (6) T1 = [1 − exp (− TR/T1*t*)], where T2*t* = 87 ms and T1*t* = 1147 ms were taken as representative relaxation times for renal tissue at 3.0 T [79]. After correlation with individual protocol parameters, correlations were computed between diffusion biomarkers and all possible products or ratios of the protocol parameters (52 combinations in all). Correlation coefficients *R* and significance levels *p* were derived for each correlation using the Igor Pro 7 software (Wavemetrics, Inc., Lake Oswego, OR USA). Significant correlations were noted both without (*p* < 0.05) and with Bonferroni correction for multiple comparisons (*p* < 0.05/52 = 0.00096). Finally, all diffusion biomarkers from healthy volunteers were grouped according to field strength (1.5 T or 3.0 T) and compared for differences with a two-tailed Student’s *t* test, for which significant differences are indicated for *p* < 0.05.

### Description of survey process

As described in the accompanying covering letter by Sourbron et al. and in keeping with the ‘approximation of a two-step modified Delphi method [80]’ for consensus building, a survey was circulated using a publicly available tool (Google Forms) to a range of renal imaging researchers with experience and/or ongoing activity in renal diffusion imaging. In addition to offering participation to all members of the PARENCHIMA collaboration, every effort was made to invite at least one researcher or corresponding author from each group contributing to the literature as surveyed previously [10]. Two rounds of surveys were circulated over a period of 4 months. Between the first and second circulation and following review of initial results with the ASL, BOLD and T1/T2 panels at a meeting in Aarhus, the list of questions was increased and refined to avoid ambiguity and increase the likelihood of reaching consensus on as many items as possible. The surveys included questions on: respondent training, patient preparation, image acquisition, diffusion parameters, analysis, and reporting. The full list of questions from the final circulation is provided in “Results” along with summarized results, percentage agreement, no basis and disagreement for all responses, as well as percentage agreement and disagreement without abstentions. Nearly, all questions tested level of agreement or disagreement qualitatively. In the first circulation, five options were provided (strongly agree/agree/neutral/disagree/strongly disagree). In the second circulation, the available responses were simplified and allowed for abstention (agree/disagree/I have insufficient experience to make a recommendation). Other questions focused on the preferred field strength or allowed multiple selections to test support of multiple related issues (e.g., reported parameters). Text comments were also collected on sets of questions of similar topics. For both rounds, responses were aggregated following the completion of the survey. The first round survey was issued on 11 January 2019, and the second on 27 March 2019; both were open for approximately 1 month.

After excluding abstentions, the level of agreement or disagreement as a percentage of all responses was calculated for each question. Responses for which either agreement or disagreement reached 75% or higher were deemed to have achieved consensus. Responses that related to one that has already reached consensus were deemed to have been resolved. For responses with agreement levels between 60 and 75%, a ‘preference’ was indicated but without the full weight of consensus. Similarly, other responses mutually exclusive from a preference or reaching lower levels of agreement on the same topic were deemed to have been resolved by that preference. Finally, the combination of all of these directly or indirectly resolved questions was considered to generate a set of recommendations.

## Results

### Literature review

**Fig. 1:**
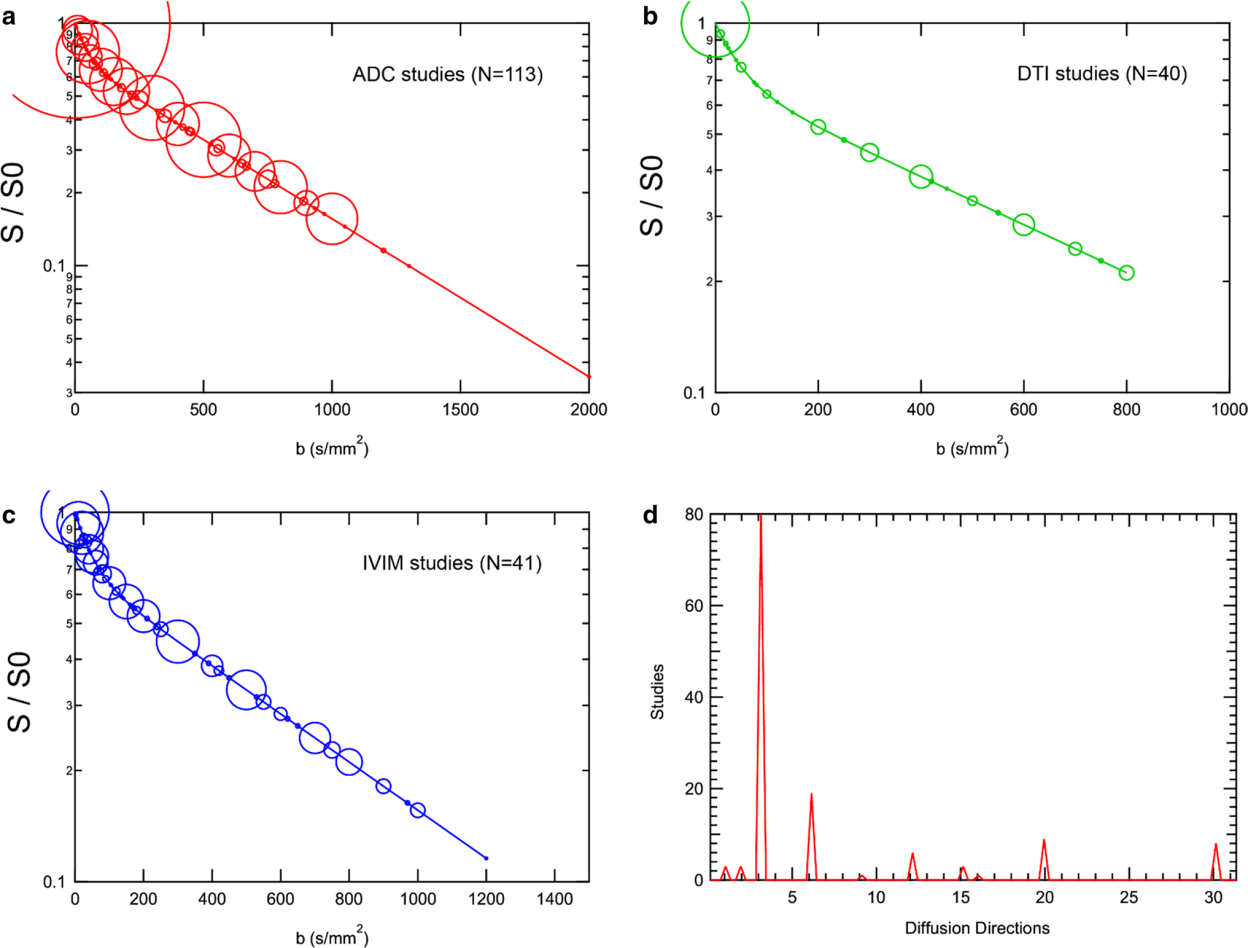
Distributions of diffusion MRI sampling in renal DWI literature studies. *b* value distributions used in studies reporting **a** ADC values, **b** DTI metrics, or **c** IVIM metrics. In the ‘bubble’ plots, the size of the circle reflects the amount of studies utilizing that *b* value. **d** Distribution of diffusion directions employed; ADC and IVIM studies dominantly employed three directions, with DTI studies employing more directions

Figure 1 shows the distributions of *b* value sampling and diffusion directions from all renal studies considered (control and patient related). Monoexponential DWI and IVIM studies have featured a continuous range of *b* values, while DTI studies have used a sparser selection, consistent with more time devoted to directional sampling. Finally, the majority of ADC and IVIM studies used three orthogonal directions for isotropic imaging. Since many of these studies employ inline processing with vendor software, the three directions are typically immediately averaged, both for convenience and for enhanced signal-to-noise ratio, to generate approximate ‘trace-weighted’ images prior to generation of ADC maps. DTI studies used six directions most often, but studies using as many as 30 directions have also been reported. While 6 directions are the bare minimum required for tensor calculation, other supplemental criteria have been suggested; for example, a minimum of 12 directions have been suggested to eliminate orientation bias in tensor results [81]. As a range of optimization studies have investigated, parameter estimation quality (both accuracy and precision) depends crucially on sufficient signal-to-noise ratio (SNR) [16, 17, 82, 83]. While thresholds and criteria vary, minimum SNR levels of 20–30 are frequently suggested for advanced renal DWI.

**Fig. 2:**
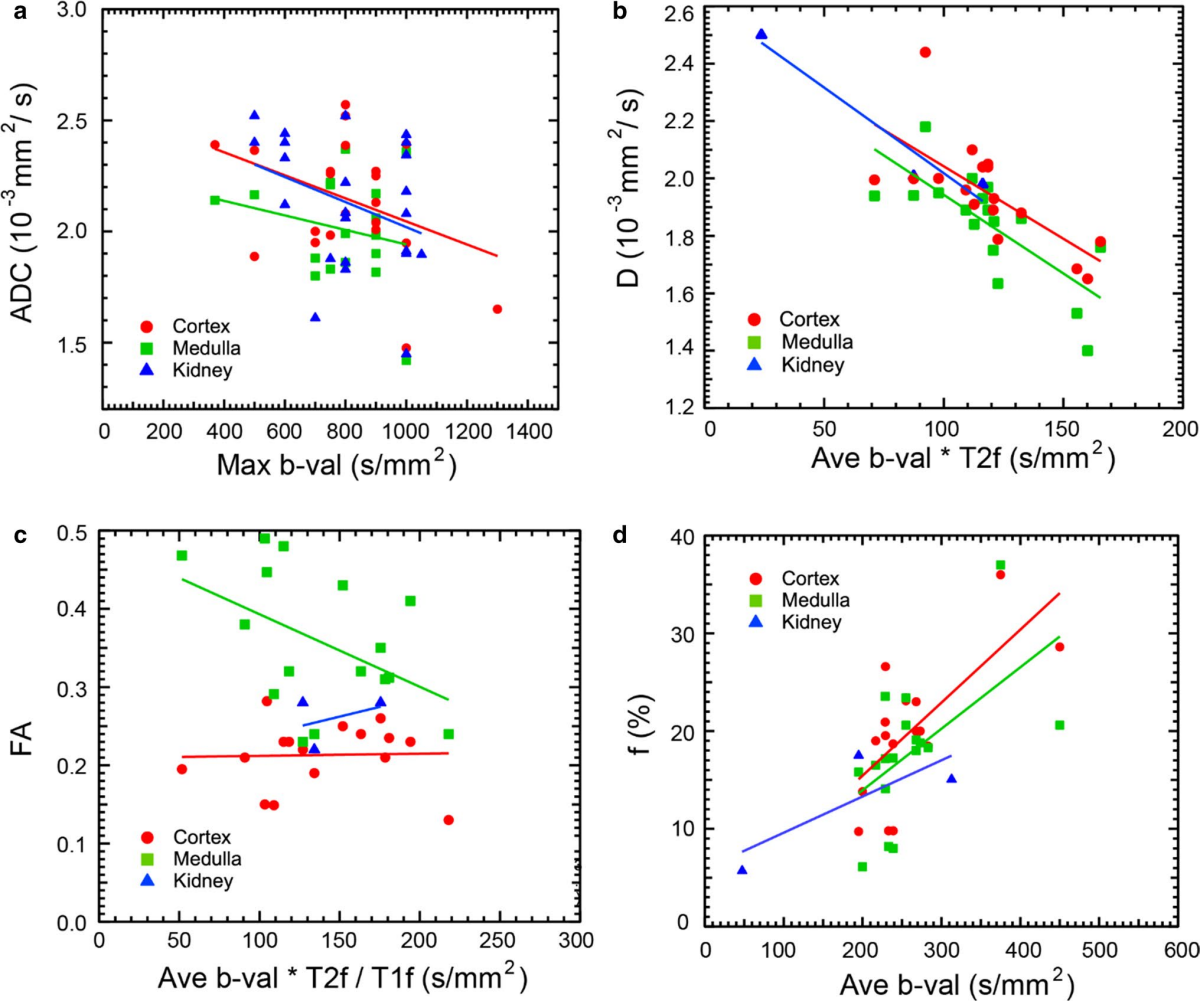
Correlations between renal diffusion MRI metrics and protocol parameters from the literature from cortex, medulla, and whole kidney tissue in healthy adults. **a** ADC, **b** IVIM tissue diffusivity *D*, **c** DTI fractional anisotropy FA, and **d** IVIM flow fraction *f* vs. average or maximum *b* value with relaxation weighting terms. Inter-study variation can be reduced when desired for larger evidence generation using more standardized protocols

Table 1 shows the results of the diffusion biomarker vs. protocol parameter correlations in healthy volunteers. All correlation results are shown for individual protocol parameters and biomarkers, and additional correlations are shown with protocol parameter combinations that provided higher correlation coefficients. The primary protocol element correlation with reported diffusion biomarkers is average *b* value, which significantly correlated negatively with tissue diffusivity *D* [Cx (cortex): *R* = − 0.506, *p* = 0.03; Md (medulla): *R* = − 0.528, *p* = 0.02] and positively with flow fraction *f* (Cx: *R* = 0.687, *p* = 0.002; Md: *R* = 0.566, *p* = 0.01). These correlations may have contributions from partial sampling of the IVIM signal response, with higher *b* value ranges providing better estimates of both slow and fast diffusion components. Conversely, if *b* values are sampled beyond the appropriate SNR level, Rician noise [84] (or more complex noise patterns accompanying image reconstruction [85]) bias can lower ADC or *D* values and inflate *f* values. Similar negative correlation trends (*p* < 0.1) are seen between cortical ADC (*R* = − 0.378, *p* = 0.08) or cortical MD (*R* = − 0.531, *p* = 0.05) and maximum b value. Transverse relaxation effects cause secondary correlations of flowing fraction with echo time TE (*R* = 0.474, *p* = 0.055) or equivalently T2 decay factor (*R* = − 0.495, *p* = 0.04), likely due to reduction of the more rapidly relaxing tissue compartment, as quantified by Lemke et al. [17], and supported by the disparate relaxation times of renal tissue [79] and serum blood [86] or urine [87–89]. Another potential modulator of contrast is diffusion time, which is lengthened at larger echo time, though the role of this parameter in renal tissue has not been conclusively mapped out. Combining *b* value and sequence timing factors together showed some amplified correlations, particularly for flow fraction and tissue diffusivity. In some cases, increasing T1 recovery increased flow representation and therefore higher f and ADC. Finally, a combination of relaxation factors and average *b* value showed a negative correlation trend (*R* = − 0.463, *p* = 0.07) with medullary FA, consistent with a modulation in flow effects on diffusion anisotropy. Figure 2 shows example correlations between renal DWI biomarkers in the literature and protocol parameters. As these variations of acquisition protocols and DWI biomarkers should be avoided in the translation of renal DWI to clinical practice, the present manuscript describes ongoing efforts to maximize lessons learned from existing work to facilitate multi-site consistency through standardized acquisition, analysis, and reporting guidelines.

**Table 1:** Correlations between reported renal diffusion metrics in the literature from cortex (Cx) or medulla (Md) regions of healthy volunteer kidneys and the corresponding studies’ protocol parameters average *b* value (ave *b* val), maximum *b* value (max *b* val), echo time (TE), repetition time (TR), T2-weighting factor (T2f), and T1-weighting factor (T1f) (see text for calculation of relaxation weighting factors)

	ADC			*D*			*f*			D*			MD			FA		
	*R*	*p*	*N*	*R*	*p*	*N*	*R*	*p*	*N*	*R*	*p*	*N*	*R*	*p*	*N*	*R*	*p*	*N*
Ave *b* val																		
Cx	− 0.162	0.47	22	− **0.506**	**0.03**	**18**	**0.687**	**0.002**	**18**	− 0.268	0.40	12	− 0.147	0.62	14	0.144	0.60	16
Md	− 0.154	0.56	17	− **0.528**	**0.02**	**18**	**0.566**	**0.01**	**18**	− 0.319	0.31	12	0.093	0.75	14	− 0.296	0.27	16
Max *b* val																		
Cx	− *0.378*	*0.08*	*22*	− 0.245	0.33	18	0.285	0.25	18	0.106	0.74	12	− *0.531*	*0.05*	*14*	0.102	0.71	16
Md	− 0.223	0.39	17	− 0.260	0.30	18	0.281	0.26	18	0.239	0.46	12	0.161	0.58	14	− 0.192	0.45	16
TE																		
Cx	0.220	0.37	19	0.149	0.57	17	*0.474*	*0.055*	*17*	− 0.262	0.44	11	0.036	0.90	14	− 0.087	0.75	16
Md	0.345	0.19	16	0.163	0.53	17	0.150	0.57	17	− 0.272	0.42	11	− 0.152	0.61	14	0.216	0.42	16
TR																		
Cx	0.225	0.44	14	− 0.270	0.30	17	0.097	0.71	17	0.392	0.23	11	− 0.168	0.57	14	0.038	0.89	16
Md	0.039	0.90	12	− 0.186	0.48	17	0.043	0.87	17	0.459	0.16	11	− 0.060	0.84	14	− 0.292	0.27	16
T2f																		
Cx	− 0.223	0.36	19	− 0.151	0.56	17	− **0.495**	**0.04**	**17**	0.249	0.46	11	− 0.083	0.78	14	0.102	0.71	16
Md	− 0.378	0.15	16	− 0.158	0.55	17	− 0.155	0.55	17	0.257	0.45	11	0.127	0.67	14	− 0.255	0.34	16
T1f																		
Cx	− 0.016	0.96	14	− 0.142	0.59	17	0.171	0.51	17	0.298	0.37	11	− 0.268	0.35	14	0.126	0.64	16
Md	0.110	0.73	12	− 0.129	0.62	17	0.018	0.95	17	0.274	0.42	11	− 0.116	0.69	14	0.079	0.77	16
Ave *b* × TE																		
Cx	0.007	0.98	19	− 0.338	0.19	17	**0.713**	**0.001**	**17**	− 0.395	0.23	11	− 0.168	0.57	14	0.086	0.75	16
Md	**0.506**	**0.046**	**16**	− 0.331	0.20	17	**0.501**	**0.04**	**17**	− 0.457	0.16	11	0.001	1.0	14	− 0.216	0.42	16
Ave *b* × T2f																		
Cx	− 0.067	0.78	19	− **0.701**	**0.003**	**17**	*0.435*	*0.08*	*17*	− 0.184	0.59	11	− 0.106	0.72	14	0.200	0.46	16
Md	0.107	0.69	16	− **0.741**	**0.001**	**17**	**0.559**	**0.02**	**17**	− 0.228	0.50	11	0.167	0.57	14	− 0.357	0.18	16
Ave *b* × T2f/T1f																		
Cx	0.262	0.37	14	− *0.479*	*0.05*	*17*	0.223	0.39	17	− 0.338	0.31	11	0.141	0.63	14	0.030	0.91	16
Md	0.175	0.59	12	− **0.503**	**0.04**	**17**	*0.427*	*0.09*	*17*	− 0.340	0.31	11	0.253	0.38	14	− *0.463*	*0.07*	*16*
Ave *b* × T1f/T2f																		
Cx	**0.691**	**0.006**	**14**	− 0.381	0.13	17	**0.682**	**0.003**	**17**	− 0.174	0.61	11	− 0.269	0.35	14	0.133	0.63	16
Md	**0.660**	**0.02**	**12**	− 0.369	0.15	17	*0.477*	*0.05*	*17*	− 0.240	0.48	11	− 0.049	0.87	14	− 0.204	0.45	16
Max *b* × T1f/T2f																		
Cx	0.110	0.71	14	− 0.155	0.55	17	**0.547**	**0.02**	**17**	0.171	0.62	11	− **0.539**	**0.047**	**14**	0.088	0.75	16
Md	0.293	0.36	12	− 0.150	0.57	17	0.310	0.23	17	0.236	0.48	11	− 0.015	0.96	14	− 0.163	0.55	16

Pearson correlation coefficients *R*, significance levels from two-sided *t* test, *p*, and number of studies contributing *N* are shown for the following diffusion parameters: apparent diffusion coefficient (ADC), IVIM tissue diffusivity (*D*), IVIM flow fraction (*f*), IVIM pseudodiffusivity (D*), DTI mean diffusivity (MD), and DTI fractional anisotropy (FA). Significant correlations (*p* < 0.05) are highlighted in bold and moderate trends (*p* < 0.1) in italics

Table 2 shows summarized diffusion biomarkers in cortex and medulla in healthy volunteers from the literature review, stratified by field strength (1.5 T or 3.0 T). The only cases showing significant differences were IVIM pseudo-diffusion (D*) and DTI mean diffusivity (MD) in cortex, both of which were higher at 1.5 T than 3.0 T.

**Table 2:** Comparisons between reported renal diffusion metrics in the literature from cortex or medulla regions of healthy volunteer kidneys at different field strengths (1.5 or 3.0 T)

	ADC	*D*	*f*	D*	MD	FA
Cortex						
1.5 T						
Mean ± SD	2056 ± 285	1966 ± 72	19.9 ± 3.2	50,800 ± 13,454	2508 ± 86	0.208 ± 0.045
*N*	12	7	7	4	4	4
3.0 T						
Mean ± SD	2243 ± 225	1919 ± 229	20.1 ± 8.4	24,964 ± 20,298	2262 ± 164	0.215 ± 0.043
*N*	10	11	11	8	10	12
*p*	0.100	0.538	0.944	**0.028**	**0.004**	0.779
Medulla						
1.5 T						
Mean ± SD	1987 ± 267	1884 ± 76	17.5 ± 5.5	57,350 ± 25,505	2348 ± 589	0.425 ± 0.079
*N*	8	7	7	4	4	4
3.0 T						
Mean ± SD	2031 ± 227	1796 ± 228	18.0 ± 7.8	29,016 ± 19,272	2092 ± 162	0.335 ± 0.082
*N*	9	11	11	8	10	12
*p*	0.721	0.261	0.877	0.110	0.452	0.105

Mean and standard deviation values, significance levels from two-sided *t* test, *p*, and number of studies contributing *N* are shown for the following diffusion parameters: apparent diffusion coefficient (ADC), IVIM tissue diffusivity (*D*), IVIM flow fraction (*f*), IVIM pseudodiffusivity (D*), DTI mean diffusivity (MD), and DTI fractional anisotropy (FA). Significant field differences (*p* < 0.05) are highlighted in bold. ADC, *D*, D*, and MD values are given in 10^−6^ mm^2^/s, *f* is given in %, and FA is unitless

### Survey results

The second-round survey included 21 respondents from 21 institutions in 8 different countries on 3 continents. 9 of 21 (43%) were radiologists, while 13/21 (57%) were physicists (11), biomedical engineers (1), or mathematicians (1). 71% of the respondents used renal diffusion for volunteer research, 76% used it for patient research, 38% used it for clinical practice, and 14% used it for clinical trials.

For the second-round survey, among the 87 questions testing levels of agreement, 23 reached consensus agreement and 18 reached consensus disagreement. These results also resolved 16 other questions on the same topics as the “parent” consensus questions. For the remaining questions, if preferences are made for 17 questions, the remaining 13 questions are resolved. The fully aggregated survey responses, as well as text comments provided, are included as supplementary material, with Table 3 summarizing results of agree/disagree questions (with those reaching consensus highlighted). Regarding magnetic field strength, a consensus majority (81%) responded either 1.5 T or 3.0 T as acceptable. Regarding reporting preferences, all suggested acquisition details (matrix, image orientation, fat suppression mode, averages, slice thickness, resolution, field of view, TR, TE, number and choice of *b* values, and number of directions) received consensus support to be reported. Reporting biomarkers in both cortex and medulla was supported by consensus. Regarding processing, motion correction algorithm, processing software, IVIM fit algorithm, and IVIM fit option received consensus support to be reported. Regarding biomarkers’ summary statistics, mean, median, and standard deviation values received consensus support to be reported. There are a range of topics that did not reach the level of consensus, including slice thickness, repetition time TR, number of signal averages, breathing mode, separate vs. combined protocols, diffusion gradient waveform, the number and highest *b* value employed, number of diffusion directions for DTI, and aspects of ROI prescriptions.

**Table 3:**
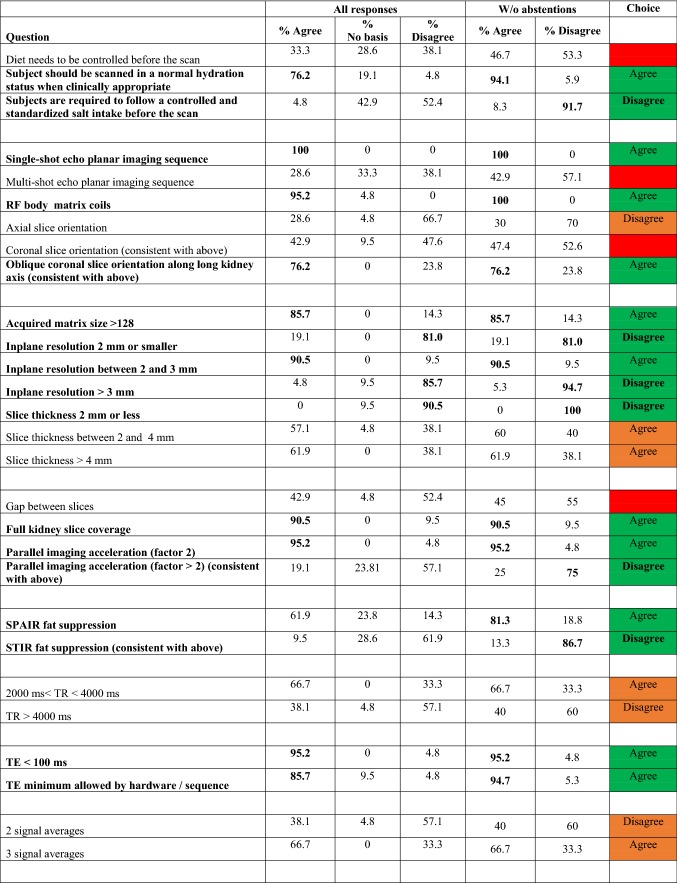

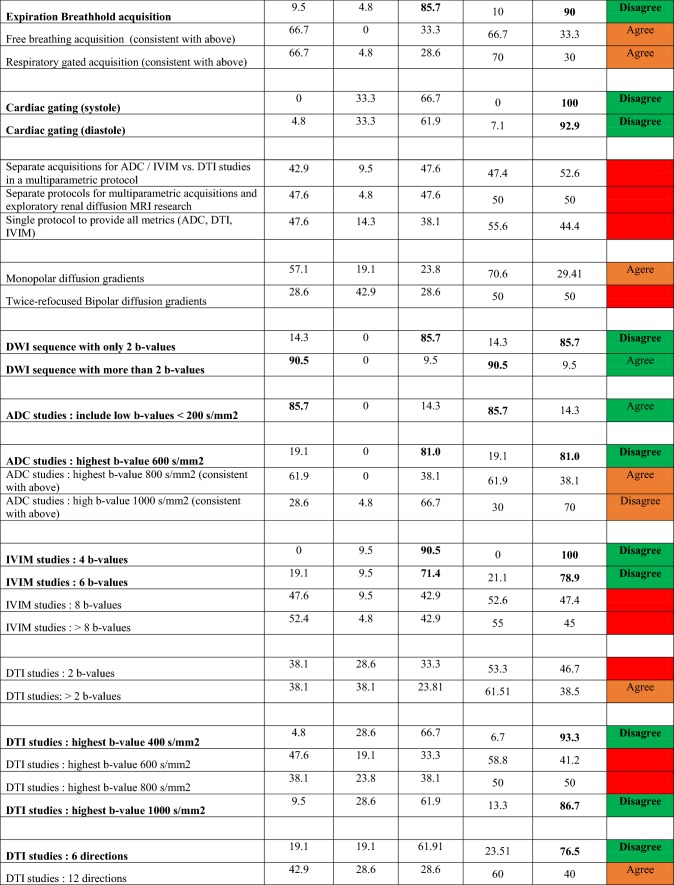

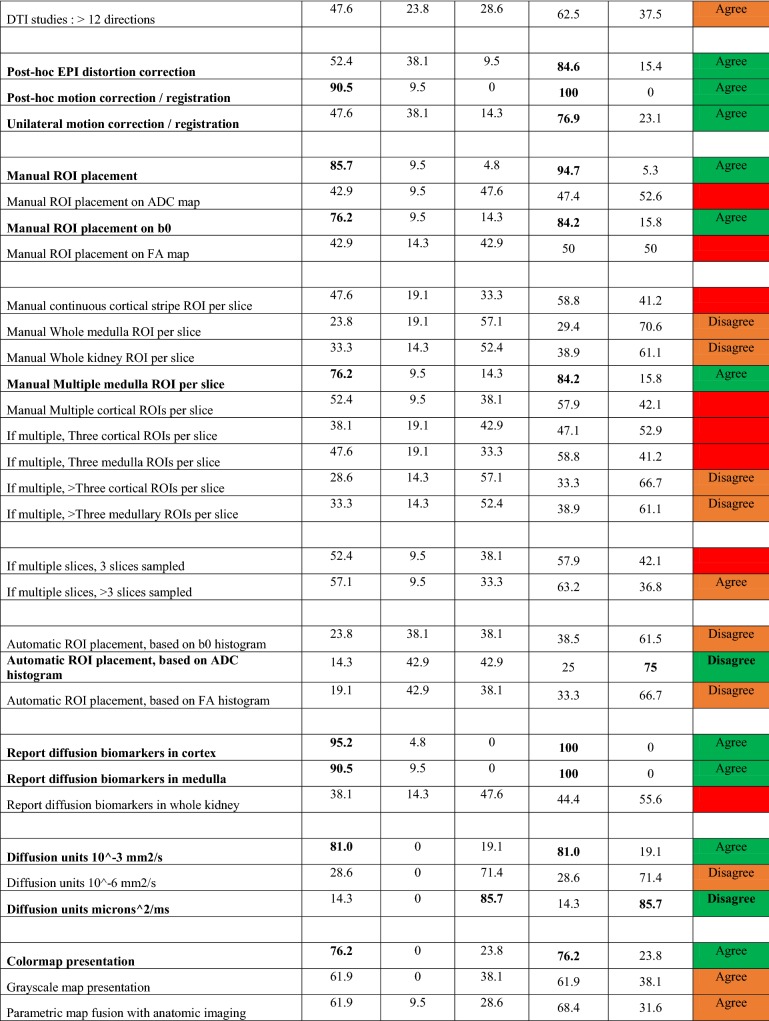
Summary of survey results on agree/disagree questions

Questions highlighted in bold achieved consensus (≥ 75%). The choice on each question (agree or disagree) is labeled and color coded; green = consensus (≥ 75%); orange = preference (≥ 60%); red = indeterminate

Considering the literature trends, consensus views, preferences, comments and practical aspects surrounding future evidence generation, recommendations are given in Table 4 for monoexponential DWI, IVIM, and DTI protocols. For many of the issues guiding protocol selection, the survey process provided clear indications of consensus choices (Table 3). For those topics not reaching consensus, we combine lesser-weighted preferences, practical issues, and information from text survey responses to synthesize recommendations. For acquisition, the consensus includes pulse sequences, RF coils, in-plane matrix/resolution, slice coverage, parallel imaging acceleration, fat suppression, echo time, and absence of cardiac gating. Strong preference (62%) was given for > 4 mm slice thickness, though some respondents expressed a desire for lower values when feasible. Strong preference (67% agreement) was also given for a TR = 2–4 s. Given some contribution of T1 weighting to parameter variability, we have suggested a standardized repetition time TR = 4 s. Breathing mode did not reach consensus; however, strong preference (70%) was given to respiratory gating and free breathing (66%). Free breathing was noted to be acceptable in cases of renal allograft imaging. We have recommended respiratory gating when available and free breathing with post hoc unilateral motion correction when not available (which was separately recommended by consensus). Regarding field strength, consensus approval for either 1.5 T or 3.0 T was found (81%), and only minimal differences were observed in the literature (Table 2). The SNR advantage of higher field is balanced by other disadvantages for DWI such as susceptibility-induced image distortion. Correspondingly, either field strength is deemed acceptable and investigators suggested to employ whichever is better equipped with hardware or software elements consistent with recommendations herein.

**Table 4:**
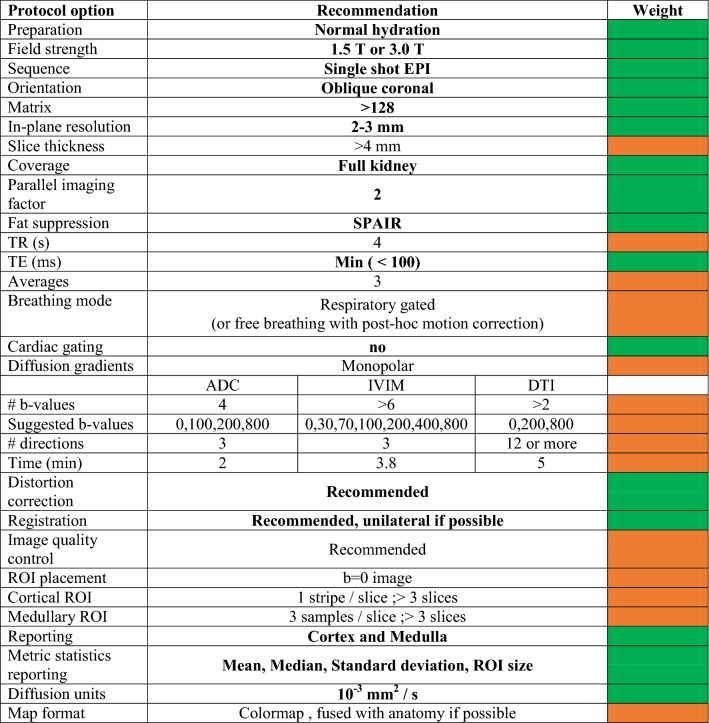
Recommendations for acquisition and processing of renal DWI data

Recommendations in bold are derived from consensus view of the expert panel. Weight of each recommendation is color coded (green = consensus (≥ 75%); orange = preference (≥ 60%)

## Discussion

The design of diffusion MRI protocols for renal imaging remains controversial, with some support for separate protocols for each diffusion technique and slightly more support for combined protocols. Similarly, separate protocols for ‘standardized’ efforts and exploratory research had only 50% support. Since deriving all measures from a combined protocol requires more sophisticated workflows than are universally available and consistent with the goals of generating generalizable evidence, we have thus recommended parsimonious protocols for monoexponential DWI, IVIM, and DTI studies. As noted below, however, the encoding parameters suggested have commonalities (e.g., *b* values) that may allow pooling of analogous biomarkers and consistency with advanced protocols involving combined encoding.

As mentioned in “Results”, field strength was not a crucial determining factor in either the diffusion metrics reported in the literature (only 2 out of 24 comparisons showed significant differences in Table 2) or in the consensus preferences of the survey respondents. The field differences observed in D* and MD in cortex in the literature values most likely arise from indirect effects of differential relaxation weighting of flow and structural compartments with field, since both have field-dependent relaxation times as discussed above. Thus, currently field strength is not a stringent requirement for standardization, although the growing technological prevalence of higher field (3.0 T) may make the point moot.

Diffusion weighting (choice of *b* values) is a crucial element of diffusion MRI protocols. For monoexponential DWI studies, consensus was found for more than 2 *b* values, including values < 200 s/mm^2^, with strong preference for a maximum *b* value of 800 s/mm^2^. For IVIM studies, consensus was found for a number of *b* values greater than 6 *b* values, with highest preference for more than 8. Finally, for DTI studies, preference was given to more than 2 *b* values (61%), with a maximum *b* value of 600 s/mm^2^ (59%). Six directions were deemed insufficient for DTI (76%), with a slight preference for more than 12 directions (63%). In addition to these indications from our panel, we may also take guidance from optimization studies on renal DWI sampling [16, 17] that emphasized the importance of several key *b* value regimes: low (0–200 s/mm^2^) intermediate (200–400 s/mm^2^), and high (600–800 s/mm^2^). Finally, we deem it valuable to suggest common encoding parameters between techniques (monoexponential DWI, IVIM, DTI) where possible to enable reasonable comparison of analogous MRI biomarkers (e.g., ADC and MD) in future datasets. Taking all of this into account, we recommend the following *b* value sets (Table 4): for monoexponential DWI studies, *b* = 0, 100, 200, 800 s/mm^2^, 3 directions; for IVIM studies *b* = 0, 30, 70, 100, 200, 400, 800 s/mm^2^, 3 directions; for DTI studies, *b* = 0, 200, 800 s/mm^2^, 12 or more directions.

Manual ROI placement had consensus support over automatic (e.g., histogram-based) placement, with the unweighted (*b* = 0) image having consensus support for ROI prescription. Cortical ROIs should be continuous stripes (one per slice), unless structural abnormalities prevent this, while medullary ROIs should be separate, with three regions sampled (upper, middle, lower poles). Generally, all slices from whole kidney coverage should be sampled with the exception of the two outermost slices where region delineation may be unclear. The consensus support for manual ROI placement is also interesting given the recent trend for machine learning (ML) and artificial intelligence (AI) in the medicine. Some efforts were made recently to adopt these techniques to renal DWI, especially in the detection of early acute renal allograft rejection [90–93]. However, great care is needed when trying to translate these approaches into the clinical arena, particularly in terms of clinical validation and measured patient-centric outcomes. We are confident that these techniques will play an important part in subsequent research studies, influence clinical translation and constitute a major focus for discussion in future versions of these recommendations.

We acknowledge some limitations in the procedures used to generate recommendations in this work. First, all entries in the literature review were assigned equal weight irrespective of population size or technological availability. Heterogeneity also exists in the survey process, in which participant elections may have been driven by different priorities and informed by different levels of clinical or technical experience. In addition, while we modeled our approach on the Delphi consensus procedure, its application was adjusted for the purposes of this review and its timeframe. The survey also highlighted other areas of disagreement between the participants. In particular, it was not possible to obtain a consensus on technical questions like the use of segmented echo planar acquisitions, or the advantage of bipolar diffusion gradients. We have not included a strategy of noise correction (pure Rician or otherwise) for more accurate quantification, but practical approaches exist that may be amenable to broad guidelines in a future iteration [85]. We also acknowledge that the imaging gradient contributions to the nominally unweighted (*b* = 0) image might lead to a potential source of error, especially in assessing *f* and D*. The full effect of this source of error has yet to be evaluated in the kidney literature. This work summarizes the large evidence base for a nonzero perfusion fraction, but the next level of standardization might refine processing to take full *b* matrices into account [94, 95].

We have also not issued a standardized prescription for phantom quality control, which has proven beneficial to DWI standardization efforts in other contexts [96, 97]; the choice of and agreement upon such a phantom for renal DWI can be revisited in the next standardization iteration. Uncertainty exists also for physiological questions such as the effect of diet on DWI. As some of these issues have already been partly addressed in the literature, the survey indicates that currently available evidence may not be sufficient for conclusive resolution. This report should, therefore, motivate a significant effort to investigate these dedicated methodological questions.

## Conclusions

The present work has summarized trends in the literature of renal diffusion MRI to date and their correlation with aspects of protocol design to direct future research efforts in the field of renal DWI. In pursuit of minimizing inter-study and inter-site variation, for the generation of evidence basis for reliable and high impact of imaging markers for renal disease, and with the guidance of a Delphi-based consensus process of experts in the field, we have generated a set of recommendations for future data collection. The recommended protocols have been chosen to be achievable by any center with clinical MRI capabilities and enable future multicentre pooling of data when equivalent protocols have been used. Therefore, these recommendations should be taken into account when starting new studies in the field of renal DWI and when reviewing submitted work in this area. We expect this recommendation process to be an iterative one and ensuing efforts may refine or add to these recommendations. To allow both growth and innovation in the field, as well as harmonization, “deviations” from these recommendations should be justified in the future studies and submissions for publication. There recommendations are intended to be updated when new evidence from ongoing or future studies is made available and change any of the recommended parameters.

Importantly, these translational efforts do not replace and are not in conflict with ongoing innovation efforts to uncover more specific biomarkers from renal DWI with more advanced methods. Instead, they reflect a view that commitment toward producing generalizable workflows in parallel will yield tremendous benefits to the field as a whole and increase chances of clinical impact on a larger scale.

## Electronic supplementary material

Below is the link to the electronic supplementary material.

Supplementary material 1 (DOCX 28 kb)
