# Correction to: Consensus-based technical recommendations for clinical translation of renal diffusion-weighted MRI

**DOI:** 10.1007/s10334-020-00828-6

**Published:** 2020-01-28

**Authors:** Alexandra Ljimani, Anna Caroli, Christoffer Laustsen, Susan Francis, Iosif Alexandru Mendichovszky, Octavia Bane, Fabio Nery, Kanishka Sharma, Andreas Pohlmann, Ilona A. Dekkers, Jean-Paul Vallee, Katja Derlin, Mike Notohamiprodjo, Ruth P. Lim, Stefano Palmucci, Suraj D. Serai, Joao Periquito, Zhen Jane Wang, Martijn Froeling, Harriet C. Thoeny, Pottumarthi Prasad, Moritz Schneider, Thoralf Niendorf, Pim Pullens, Steven Sourbron, Eric E. Sigmund

**Affiliations:** 1 Department of Diagnostic and Interventional Radiology, Medical Faculty, University Dusseldorf, Moorenstr. 5, 40225 Düsseldorf, Germany; 2 Department of Biomedical Engineering, Istituto di Ricerche Farmacologiche Mario Negri IRCCS, Bergamo, Italy; 3 MR Research Centre, Department of Clinical Medicine, Aarhus University, Aarhus, Denmark; 4 Sir Peter Mansfield Imaging Centre, University Park, University of Nottingham, Nottingham, NG7 2RD UK; 5 Department of Radiology, Cambridge University Hospitals NHS Foundation Trust, Addenbrooke’s Hospital, Cambridge, UK; 6 Translational and Molecular Imaging Institute and Department of Radiology, Icahn School of Medicine at Mount Sinai, New York, NY USA; 7 Developmental Imaging and Biophysics Section, UCL Great Ormond Street Institute of Child Health, London, UK; 8 Imaging Biomarkers Group, Department of Biomedical Imaging Sciences, University of Leeds, Leeds, UK; 9 Berlin Ultrahigh Field Facility (B.U.F.F.), Max Delbrueck Center for Molecular Medicine in the Helmholtz Association, 13125 Berlin, Germany; 10 Department of Radiology, Leiden University Medical Center, Leiden, The Netherlands; 11 Department of Diagnostic, Geneva University Hospital and University of Geneva, 1211 Geneva-14, Switzerland; 12 Department of Radiology, Hannover Medical School, Hannover, Germany; 13 Die Radiologie, Munich, Germany; 14 Department of Radiology, Austin Health, The University of Melbourne, Melbourne, Australia; 15 Department of Medical Surgical Sciences and Advanced Technologies, Radiology I Unit, University Hospital “Policlinico-Vittorio Emanuele”, University of Catania, Catania, Italy; 16 Department of Radiology, Children’s Hospital of Philadelphia, Philadelphia, PA USA; 17 Department of Radiology and Biomedical Imaging, University of California San Francisco, San Francisco, CA USA; 18 Department of Radiology, University Medical Center Utrecht, Utrecht, The Netherlands; 19 Department of Radiology, Hôpital Cantonal Fribourgois (HFR), University of Fribourg, 1708 Fribourg, Switzerland; 20 Department of Radiology, Center for Advanced Imaging, NorthShore University Health System, Evanston, IL USA; 21 Department of Radiology, University Hospital, LMU Munich, Munich, Germany; 22 Comprehensive Pneumology Center, German Center for Lung Research, Munich, Germany; 23 Ghent Institute for Functional and Metabolic Imaging, Ghent University, Ghent, Belgium; 24 Department of Radiology, University Hospital Ghent, Ghent, Belgium; 25 Department of Radiology, Center for Biomedical Imaging (CBI), Center for Advanced Imaging Innovation and Research (CAI2R), NYU Langone Health, New York, NY USA; 26 Department of Radiology, University Hospital Tuebingen, Tübingen, Germany

## Correction to: Magnetic Resonance Materials in Physics, Biology and Medicine 10.1007/s10334-019-00790-y

The article Consensus-based technical recommendations for clinical translation of renal diffusion-weighted MRI, written by Alexandra Ljimani, Anna Caroli, Christofer Laustsen, Susan Francis, Iosif Alexandru Mendichovszky, Octavia Bane, Fabio Nery, Kanishka Sharma, Andreas Pohlmann, Ilona A. Dekkers, Jean-Paul Vallee, Katja Derlin, Mike Notohamiprodjo, Ruth P. Lim, Stefano Palmucci, Suraj D. Serai, Joao Periquito, Zhen Jane Wang, Martijn Froeling, Harriet C. Thoeny, Pottumarthi Prasad, Moritz Schneider, Thoralf Niendorf, Pim Pullens, Steven Sourbron and Eric E. Sigmund, was originally published electronically on the publisher’s internet portal on 01 November 2019 without open access. With the author(s)’ decision to opt for Open Choice the copyright of the article changed on 10 January 2020 to © The Author(s) 2020 and the article is forthwith distributed under a Creative Commons Attribution 4.0 International License (https://creativecommons.org/licenses/by/4.0/), which permits use, sharing, adaptation, distribution and reproduction in any medium or format, as long as you give appropriate credit to the original author(s) and the source, provide a link to the Creative Commons licence, and indicate if changes were made.

