# Supplementary material for: Consensus-based technical recommendations for clinical translation of renal diffusion-weighted MRI
Source: MAGMA. 2019 Nov 1;33(1):177–95. doi: 10.1007/s10334-019-00790-y (PMC7021760; doi:10.1007/s10334-019-00790-y)
Supplement: Supplementary file 1 — Supplementary material 1 (DOCX 28 kb) [file 10334_2019_790_MOESM1_ESM.docx]

**Comments (diet)**

We use it along with other techniques like BOLD and ASL and so we always prefer to use at least 4 hr fasting.

It may help to reduce susceptibility artifacts from bowel gas if subjects avoid large meals, fiber, red meat 24 hrs before scan.

Hydration

Avoiding a high protein diet before the scan and having light meals 24 hours before the examination would be helpful (this would also reduce colonic loading that might interfere with the MRI acquisition).

Not possible in daily routine.

DWI may be frequently performed along with other techniques such as BOLD which will require some standardization of diet/water intake.

**Comments (hydration)**

Hydration has an influence over diffusion parameters.

Normal hydration level (1 glass water) suggested

Standard hydration should be preferred

This should be guided by eliciting clinical history on the day of the scan - anything between 250 - 500 ml of water would be appropriate if no clinical contra-indications (such as existing oedema, etc).

**Comments (salt intake)**

Seems difficult to control, would need a very specific list of allowed/forbidden foods and quantities.

This would be difficult to achieve for patients that are not already on a controlled diet and the non-compliance rate would be high (as well as potentially result in failures of patient recruitment).

**Comments (k-space readout)**

I have always used single-shot EPI

Speed is an issue with multi echo shot EPI

This should be guided by the information available in the current literature that yields best image quality.

**Comments (field strength)**

Higher distortion in coronal at 3T

3T preferred when available.

**Comments (image orientation)**

Coronal. Oblique not mandatory but definitely an option in particular if simplifies matching geometry to other MR acquisitions as part of a multiparametric protocol. The TRs are normally long enough to allow full kidney coverage even if doing pure coronal (i.e. no oblique).

As part of multiparametric approach, it makes sense to have all the acquisitions in the true coronal plane.

Axial minimizes distortion, but coronal to scanner or the kidney matches the other sequences in an multiparametric MRI protocol.

Long axis alignment (using anatomic referencing) best chance for standardized regional sampling

Oblique coronal slice orientation along long kidney axis (for native kidneys), axial plane for transplanted kidneys

In-plane motion preferred

Ideally the DWI acquisition should match and be "mapped" to anatomical imaging / other MRI pulse-sequence acquisitions so that renal mpMRI protocols become. reality.

We do axial DWI and coronal DTI. In our experience, for quantitative imaging, straight axial and straight coronal works well with consistency in clinical practice. With oblique, there is potential to automatically increase TE if FOV gets changed due to patient size or if the angle gets steep.

Oblique coronal slice orientation along long kidney axis should be done with two independent slice packages, one aligned to the left and one aligned to the right kidney.

I think would be reasonable to acquire both axial and oblique coronal.

In my opinion all orientations can be used.

**Comments (image matrix)**

Depends on FOV

agree, with lower than 128, the spatial resolution is too low.

**Comments (inplane resolution)**

High resolution (2mm or smaller) would increase the acquisition time and the noise; low resolution (>3mm) would be not enough.

SNR is an issue with high inplane resolution.

**Comments (slice thickness)**

[4-5] mm

Ideally it would be great to have slice thickness between 2 and 4mm, but taking into account the time limitation even 5-6mm could be enough (I vote for saving time using higher slice thickness and use it for acquiring more b values).

Higher SNR, more kidney coverage in fewer slices and less acquisition time.

Large slice helpful for sufficient SNR in advanced protocols.

SNR again an issue.

Slice thickness>4 mm increases SNR

We make every effort to use 3mm in general. although 4 mm has been occasionally used clinically for speed if running out of time.

**Comments (slice coverage)**

More than 1 slice, e.g. 3 slices.

**Comments (parallel imaging)**

I have always used factor = 2 - no experience with factors>2

For EPI, parallel imaging will reduce effective TE and potentially reduce artifacts

If sufficient snr eg at 3t

factor>2 makes images noisy with the current acquisition method. perhaps something to reconsider in future, in combination with SMS or CS.

2 or higher

**Comments (fat suppression)**

Is an option for a spectrally selective method missing? I normally use that at 3T with acceptable results (i.e. the so called FatSat on Siemens)

I have always used SPAIR fat suppression (called ASPIR on GE scanners)

With EPI, fat suppression is essential

**Comments (TR)**

Collected respiratory triggered

[4-5] s

TR depends also by respiratory triggered acquisition

5100 ms

**Comments (TE)**

70 ms

Should be guided by hardware capabilities.

as long as it is consistent.

**Comments (signal averages)**

Depends on no. directions / scan time / bval. If time allows do multiple averages particular at the highest bvals. Can also allow retrospective rejection of corrupted volumes (e.g. dropout)

It's again a matter of acquisition time, as having 2 averages doubles the acquisition time. We prefer to use just 1 average and acquire a higher number of b values

Even more if using free breathing

More signal averages for higher b-values

NSA>3 increases SNR ratio

1

Ideally the acquisition should be good enough to allow good quality data to be acquired from a single acquisition. if absolutely;y necessary 2 signal averages should be the maximum.

**Comments (breathing modes)**

I would use resp. gating or triggering if scan time allowed.

A breathhold is not feasible for long IVIM acquisitions. Free breathing saves time and is ok for allografts, navigator-gated was shown in a previous study to be better at controlling motion in native kidneys. Co-registration if data is necessary with either, anyway.

For extended advanced protocols, free breathing would be preferred along with image registration; similarly if respiratory gating is not available

free breathing for transplanted kidneys

If patient well-trained and co-operative respiratory gating would be a reasonable option. Expiration breath-hold would prolong the acquisition time for too long (for whole kidney coverage) with the risk of motion artefacts / corrupted data.

FB helpful if possible in future.

Respiratory gated acquisition would be best but often impractical

all three can be used

**Comments (cardiac gating)**

Never used for DWI

informative for research studies but for multiparametric MR may be time prohibitive

For advanced / research applications only - not enough data available in the area for confident assessment. However, it would make sense, in the future, to have this incorporated.

have not explored the cardiac gating if it makes a difference

**Comments (separate vs. combined protocols)**

If you combine protocols you save a lot of time

Ideally, there should be a single protocol for all metrics, although it may be more difficult to implement. Protocols for mpMRI and exploratory diffusion research can be different, but the goal should be to integrate the result of the exploratory research into mpMRI.

Single protocol time-efficient but requires specialist processing

if possibile all metrics in a single protocol

Provided that the focus is a DWI / DTI / IVIM acquisition - it is difficult to see how other more advanced imaging sequences could be fitted in in addition to this protocol and anatomical imaging (maybe BOLD / T1 mapping could be "squeezed in").

Preferably one single protocol to provide all metrics for both exploratory DWI reserch, and multiparametric acquisitions

**Comments (DWI number of b-values)**

I mean 2 non-zero b-values in addition to the b=0 s/mm2 non diffusion-weighted baseline scan (having a relatively simple clinical protocol in mind)

At least 3 b-values samples the exponential curve and provides better ADC values.

For IVIM severla low b-values (below 100) are needed

min. 3 per directions

**Comments (ADC low b-value inclusion)**

Ok to see the perfusion effect on ADC. Can do IVIM if we want to separate the diffusion effect from perfusion.

allows some sensitivity to flow effects which are typically relevant; helpful to include even if ADC only is collected

for pseudodiffusion

**Comments (ADC highest b-value)**

limited by gradient hardware

I am happy with highest on the range of [600-800] s/mm2 but having to choose only one for consistency I went with 800 s/mm2

Values above 1000 would need DKI fit of signal.

700-1000

b=800 seems to be sufficient for kidneys. b1000 images look noisy.

**Comments (IVIM number of b-values)**

Low number of b values makes it impossible to use a segmented fitted approach (which seems to me the best option)

Currently using 9 b-values, have not performed a study to see if fewer can be used.

8 samples should be sufficient once IVIM model has already been selected for use

If one is interrested in the perfusion fraction f solely (not in D*), 6 b-values might suffice.

the more b-values (especially low ones) the better, however problem of time in daily routine

considering possible 3 diff. components including tubuli

**Comments (DTI number of b-values)**

I mean 2 non-zero b-values in addition to the b=0 s/mm2 non diffusion-weighted baseline scan (having a relatively simple clinical protocol in mind)

I have never performed DTI

a minimal set that would allow comparison of diffusivities from other protocols

**Comments (DTI highest b-value)**

I am happy with highest on the range of [600-800] s/mm2 but having to choose only one for consistency I went with 800 s/mm2

**Comments (DTI number of directions)**

Dependent on scan time and number of averages in each direction. I would recommend 12 as a minimum

6 is the minimum, the more the better, with acquisition time as the limitation.

a compromise of time and SNR

6 Directions for the fractional anisotropy, tractography likely requires more.

**Comments (image quality control)**

Distortion correction would be helpful in some cases but not sure distortion correction tools are mature enough for renal clinical applications so probably would not recommend using it as the default approach. Unilateral motion correction seems mandatory to me if doing rigid registrations as the kidneys do move independently to a significant extent.

Unilateral correction more difficult to be performed

Not sure what is meant by unilateral motion correction: for 1 kidney only?

unilateral correction essential (in free breathing protocol) given independent motion

QA / QC should be implemented at acquisition level in the first instance so that the radiographers are well trained and recognise if an acquisitions needs to be repeated to achieve high quality data.

**Comments (map for manual ROIs)**

Recommending a method for ROI choice is not trivial. If the cortico-medullary differentiation is still possible (may depend on CKD stage) AND data has been properly acquired and motion-corrected, the FA maps provide a good source of contrast to readily differentiate cortex and medulla. If not, I would resort to drawing in the b0 map.

Rather place ROI on b0 rather than maps, to avoid artifacts.

FA map useful for medulla selection when DTI is available

Why not semi-automatic? I think we are still quite far from automatic ROI placement.

**Comments (shape of manual ROIs)**

Continuous cortex and multiple medulla ROIs as default. The exception would be if cysts or other structural abnormalities exist which would not allow one to draw a continuous cortical ROI.

Using multiple ROIs rather one large one allows avoidance of artifacts. Cortex and medulla ROI should be separated, as they have different vascularisation and physiological properties. Although there is poor contrast to differentiate Cx from

Med on diffusion, anatomical images can be used as reference.

large ROIs best use of available data

Medulla-wedge shaped, cortical curvilinear

Circles or any other shape as long as they confidently select anatomically-correct parts of the cortex / medulla. Why not use anatomical imaging, well-registered with ethical DWI / ADC / etc, with good cortico-medullary contrast to guide this process?

Shape of the manual ROIs should aline with the anatomicl shape of cortex and medull

**Comments (slices for ROI sampling)**

More is better but drawing ROIs manually is a laborious process so I'd say sampling from 3 slices not a dealbreaker.

Depends on the slice thickness.

In my case I use 5mm thickness + 1 mm gap; taking 3 slices (central + slice before and after) you already have a representative portion of the cortex and medulla.

For whole kidney I would suggest to remove the first and last slice and take all the others

**Comments (automatic ROI placement)**

Most automatic methods depend on cortico-medullary differentiation existing. In patients this may be an issue. However as mentioned above where this exists the FA maps provide a good source of contrast.

dangerous - highly affected by artefacts

Automatic ROI placement might become possible using AI methods in the future. Presently, I don't think automatic approaches based on histograms are reliable.

supervised ROI placement is essential. Automatic may be attempted if software allows. Our software doesn't allow automatic yet. we are working on developing an automated version.

**Comments (acquisition details reporting)**

Other things to report: scanner vendor/model; field strength; fat suppression used (yes or no at least); any physiological triggering/gating if used; bandwidth; echo-spacing; slice gap if used; order of acquisition of slices (sequential/interleaved); if undersampling was used (either partial Fourier and/or parallel imaging). This may sound like a lot, but it is generally hard to replicate protocols from papers if they are not comprehensive.

parallel imaging factor, receiver bandwidth

Maybe all these parameters should be "assembled" as a guide to reviewers and be a "requirement" for future submissions in the field (that could be adopted by journals, such as JMRI / MRM).

Most of these specific acquisition details are quite general, and might be more suited for the general PARENCHIMA paper as discussed in Aarhus.

**Comments (processing details reporting)**

in addition: ROI used (whole kidney/cortex/medulla vs multiple ROIs, manual vs automatic ROI)

Do not have experience with ivim

Most of these specific postprocessing details are quite general, and might be more suited for the general PARENCHIMA paper as discussed in Aarhus.

**Comments (regional metrics reporting)**

Like to separate cortex from medulla

As many metrics as possible should be reported - this is the only way in which the best parameters / biomarkers will emerge as potential candidates for clinical translation.

**Comments (diffusion units reporting)**

microns^2/ms can make the text cleaner but not standard so may still confuse people especially as bvals almost always referred to as s/mm^2

I do not use smaller units, but they may be better as they would highlight differences in ADC or D without having to use many decimals.

**Comments (summary statistics reporting)**

Elaborate on single-subject vs. group metrics (e.g. stdev)

DWI biomarkers often have non-normal distribution

Partly depends on the distribution of the data

**Comments (parametric map presentation)**

Grayscale seems to be standard especially for FA, but if using colorscale use perceptually linear ones (not jet!). Fusion can be nice but would not recommend as the default approach.

Maps and maps + anatomy fusions are great for presentation purposes.

fusion challenging given EPI distortion; grayscale most standardizable

This depends on the local preference, looks nice, but no real benefit
